# Hysterical Amaurosis

**Published:** 1890-01-18

**Authors:** 


### The Practitioner's Outlook and Retrospect.

[*The Editor will be glad to receive offers of co-operation and contributions from members of the profession. There is no doubt that much valuable material full of interest and instruction is at present lost to science. This is largely so in the case of* (1) *the younger and less-known members of the hospital staff,* (2) *those connected with cottage hospitals, and* (3) *the great body of medical practitioners not attached to any institution. The co-operation of all is cordially invited to enable the Editor to make* The Hospital *of the utmost possible use and value to the profession. Specialists willing to review books on their special subjects are invited to communicate this fact at once. All letters should be addressed to* The Editor, The Lodge, Porchester Square, London, W.]

### MEDICINE.

### HYSTERICAL AMAUROSIS.

Mr. James Milner, of Shipdham, Norfolk, details in the *Lancet* of December 7 a curious case of hysterical amblyopia. A girl, æt. sixteen, complained of loss of vision which came on gradually. The lids were closed and inability to open them professed, but there was spasmodic twitching. On forcibly opening the lids, nothing whatever amiss could be found in the eyes, although the girl declared she could not see, and did not flinch from flame held close to the eyes, Mr. Milner's first idea was temporarily to impair vision, so as to actually cause some anxiety in the patient's mind. This was done by atropine. Afterwards electricity was used, with partial success. The girl was then spoken to as to the foolishness of her conduct. Suddenly, under strong faradaic current, the girl opened her eyes widely, burst into tears, and the case was at an end. There was no menstrual irregularity, no mental distress, and the girl was in a comfortable situation. But some of the children had been obliged to wear glasses, and this was the only cause Mr. Milner could find to account for his patient's attack of false amaurosis.

